# Phytoremediation potential of switchgrass (*Panicum virgatum)*, two United States native varieties, to remove bisphenol-A (BPA) from aqueous media

**DOI:** 10.1038/s41598-019-56655-w

**Published:** 2020-01-21

**Authors:** Jacob C. Phouthavong-Murphy, Alyssa K. Merrill, Stephanie Zamule, David Giacherio, Beverly Brown, Carol Roote, Padmini Das

**Affiliations:** Nazareth College of Rochester, 4245 East Ave, Rochester, NY 14618 USA

**Keywords:** Pollution remediation, Environmental impact

## Abstract

Plastic wastes burdening Earth’s water and accumulating on land, releasing toxic leachates, are one of the greatest global threats of our time. Bisphenol-A (BPA), a potent endocrine disrupting compound, is a synthetic ingredient of the polycarbonate plastics and epoxy resins used in food containers, cans, and water bottles. Bisphenol-A’s rising concentrations in the environment require a sustainable alternative to current removal practices, which are expensive and/or ecologically unsafe. Switchgrass offers a safe alternative. To investigate its potential for BPA removal, two United States native switchgrass varieties where tested in hydroponic media. Results show minimal hydrolysis and photolysis of BPA over 55 days, confirming its persistence. Both generic and heavy metal switchgrass exhibited statistically significant (p < 0.0001) BPA removal (40% and 46%, respectively) over approximately 3 months, underscoring switchgrass’s effectiveness for BPA removal. Significantly higher (p < 0.005) BPA accumulation in roots than shoots and nonsignificant variances in biomass, chlorophyll (p > 0.19), and peroxidase between BPA-treated and untreated plants indicates substantial BPA tolerance in both varieties. Kinetic parameters of BPA removal and translocation factors were also determined, which will inform the design of BPA removal phytotechnologies for a variety of soil conditions, including landfills where BPA accumulation is greatest.

## Introduction

Discovered in the 1890s, as a potential drug, 4,4′-(propane-2,2-diyl)diphenol (bisphenol-a, BPA) was later found to be useful in plastic production^1^. Manufacturing companies use large quantities of BPA in polyacrylamide, which is distributed to the public in food containers, baby bottles, and a variety of other consumer products^2^. In 2012, worldwide BPA production reached an estimated 6.5 million tons per year, with predicted increases of 4.6% annually until 2019^3,4^. In 2015, an estimated 7.7 million tons of BPA were produced, representing an even greater increase (6% per year) than initially predicted^5^. By the end of 2022, production is estimated to be 10.6 million tons^5^. Products containing BPA do not remain in use indefinitely, it is estimated over 450 tons of BPA are released into the environment annually^6^.

In 2012, the United States (U.S.) Food and Drug Administration banned BPA in infant products due to controversy over developmental toxicity risks^7,8^. However, BPA is still utilized in the production of a variety of plastics for other consumer uses, including polyvinyl chloride, polycarbonate, and thermal receipt paper^9^. Despite its widespread use, BPA has been linked to numerous adverse health outcomes, including infertility, tumor growth, and obesity^10–12^. Bisphenol-A exposure occurs, since the potential exists for BPA to leach out of disposed BPA containing products into the environment through degradation, posing a risk to humans who accidentally consume BPA through food and/or drinking water and organisms who come into contact with polluted areas^13–15^.

Cellular function disruption due to BPA has been reported at levels as low as 0.23 ng/L, with the solubility of BPA approximately 100 mg/L^16^. A National Health Survey conducted by the U.S. Center for Disease Control and Prevention found over 95% of human urine had BPA levels above 10 ng/L^16,17^. At present, no maximum permissible concentration of BPA in water has been established by the U.S. Environmental Protection Agency, though the intake limit is set at 0.05 mg/kg/day^6^. As the production of plastics containing BPA continues to rise, the environmental concentrations of BPA most likely will increase concurrently. With landfill leachate concentrations presently documented as high as 17 mg/L, exposure to dangerous levels of BPA is expected to escalate as more BPA containing products are released into the environment^18,19^.

While the potential exists for degradation of environmental BPA through hydrolysis or photolysis, the quantity of BPA eliminated through degradation in pure water is not significant^4,20,21^. Rather degradation occurs only in the presence of humic substances, due to reactive oxygen species found in conjunction with humic substances^22^. A previous study found the half-life of BPA in surface water ranged from 66 hours to 160 days from photolytic degradation, due to water condition variation^4^. Further, the degradation observed over 160 days did not prevent BPA from traveling through the water system and harming aquatic organisms^4^. Thus, additional methods of BPA removal must be found.

Researchers seeking methods to remove environmental BPA face a multitude of challenges. Widely utilized remediation techniques are expensive and not ecologically viable^23^. Chemical degradation using nanotechnology, TAML (Tetra-amido macrocyclic ligand)/H_2_O_2_ complex and other methods have demonstrated only minimal success, and the potential toxicity of these chemicals on the environment is a concern^24,25^.

Phytoremediation shows promise as a sustainable alternative to remove BPA from contaminated soil and water systems in a cost-effective, ecologically-friendly, and socially-acceptable way^26^. Phytoremediation has been successfully utilized for the removal of a number of other environmental contaminants, including tetracycline, polychlorinated biphenyl, and trinitrotoluene^27–29^. Organisms previously shown to take up BPA include aquatic plants: *Azolla filiculoides* (water velvet)*, Potamogeton illinoensis* (Illinois pondweed), *Lemna* species (duckweed), *Spirogyra* (algae); terrestrial plants: *Portulaca oleracea* (common purslane), *Dracaena sanderiana* (lucky bamboo), *Fragarua vesca* (wild strawberry), *Vicia faba* (broad bean), *Oryza sativa* (Asian rice); and fungi: *Fusarium sporotrichioides*, *Fusarium monoiliforme*, *Aspergillus terreus*, and *Aspergillus nidulans*^30–38^. Although a number of plants capable of up taking BPA have been identified, few meet the criteria for a landfill phytoengineering technique required in the U.S.

Environmental BPA levels are much higher in landfill leachate (17 mg/L) compared to surface water (12 µg/L), making it essential to develop remediation technologies to extract BPA from landfills, thus preventing further leaching into the surrounding soil and groundwater, and greater exposure to organisms^6,19^. Phytoextraction using a BPA-tolerant plant offers a promising remediation method, however, selecting an appropriate plant species to be applied in a landfill environment poses a number of challenges. Native varieties are preferable to avoid risks to environmental health caused by invasive species. Edible plants should be bypassed to reduce the risk of further accumulation of BPA in the food chain. The plant should be terrestrial and capable of tolerating other environmental contaminants frequently present in landfill conditions. Prior to implementation, the non-edible, terrestrial plants native to the area of interest should be thoroughly studied to ascertain the plant’s ability to tolerate BPA toxicity and remove BPA from contaminated systems.

Thus a phytoengineering technique using native, non-edible plants to extract BPA from landfills in the U.S. must be developed. Most BPA-tolerant plants currently reported are either aquatic, edible, or not native to the U.S.^33,35,36,39^, and therefore do not satisfy all criteria for successful landfill application in the U.S. The search for a suitable plant capable of thriving in landfill conditions in the U.S. led to the study of switchgrass (*Panicum virgatum*): a terrestrial, perennial, high biomass grass, native to all of the U.S., except California and the Pacific Northwest^40^. Current uses of switchgrass include erosion control, wild life habitat, fodder, ornamental purposes, paper pulp, and biofuel^41^. Switchgrass has been previously shown to remove heavy metals including lead, cadmium, zinc, chromium, as well as organic contaminants like atrazine^42–44^. The phytotolerance of switchgrass to a variety of toxic contaminants adds promise for switchgrass’ potential survival in contaminated soils and landfills, where a wide range of pollutants are frequently present in variable concentrations. Additionally, switchgrass is hardy and capable of surviving in a wide range of soil types and climates^41^.

The present investigation aimed to compare the relative potential of two switchgrass varieties to extract BPA from aqueous media at a concentration relevant to the maximum BPA concentrations reported in U.S. landfills. This appears to be the first attempt to investigate the potential of BPA removal using switchgrass, whose effectiveness in removing other toxic chemicals from contaminated systems and phytotolerance against them has been documented^42–44^. Based on previous research, both varieties of switchgrass were expected to remove a significant amount of BPA from an aqueous media and translocate it into the stalk, making switchgrass a suitable solution to environmental BPA pollution. Investigating the relative effectiveness of BPA removal by two varieties of one plant species is also a novel approach in phytoremediation and will contribute valuable insight to implementation, as some varieties of one plant could be native to one geographical area and others may not. The kinetics of BPA removal from contaminated hydroponic media over almost three months, the accumulation of BPA in different plant tissues, and the phytotoxic effects of BPA on switchgrass were also evaluated. This study is an initial step toward achieving the long-term goal of designing a phytoengineered technique for BPA removal from landfill environments.

## Results

### Hydrolysis and photolysis

**Figure 1:**
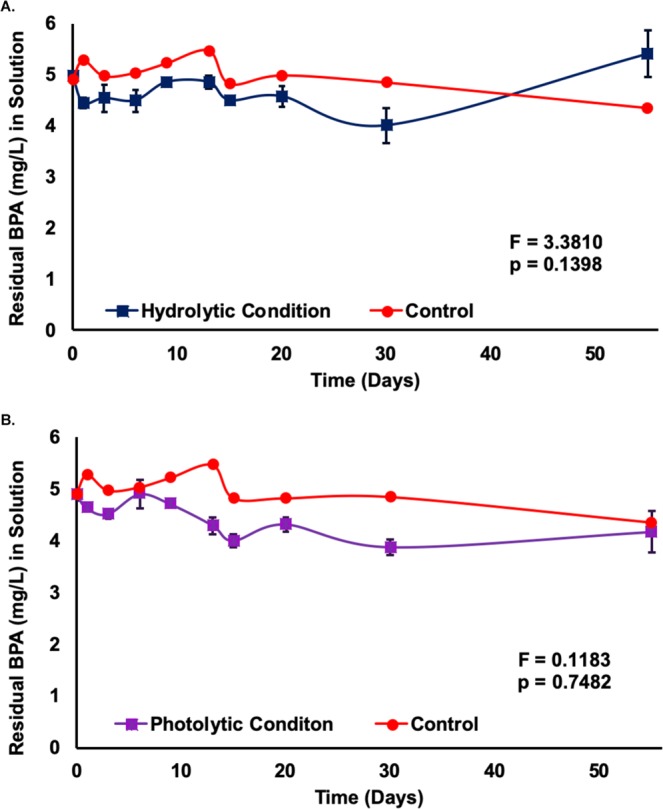
Residual BPA (mg/L) in Aqueous Media under Hydrolysis (**A**) and Photolysis Conditions (**B**). (**A**) Data are expressed as mean (n = 3) ± one standard deviation. Mean comparison was carried out by Tukey-Kramer HSD Test. (**B**) Data are expressed as mean (n = 3) ± standard deviation. Mean comparison was carried out by Tukey-Kramer HSD Test.

Hydrolysis and photolysis of an environmentally relevant (5 mg/L) concentration of BPA solution without plants were tested in the absence (hydrolysis) and presence (photolysis) of light, respectively, resulting in minimal BPA degradation (hydrolysis = 0%, photolysis = 14.9%, control = 11.4%). No significant differences (hydrolysis p = 0.1398, photolysis p = 0.7482) were detected between control and hydrolytic/photolytic samples over the experimental duration of 55 days (Fig. 1), demonstrating BPA does not significantly degrade when exposed to hydrolytic and photolytic conditions.

### BPA removal from aqueous media by *panicum virgatum*

**Figure 2:**
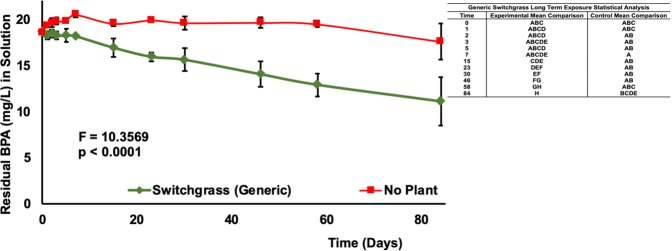
Residual BPA (mg/L) in Aqueous Media at 20 mg/L Initial BPA Concentration over a Period of 85 Days with Switchgrass (Generic). Data are expressed as mean (n = 3) ± one standard deviation. Mean comparison was carried out by Tukey-Kramer HSD Test and shown in the indented table.

**Figure 3:**
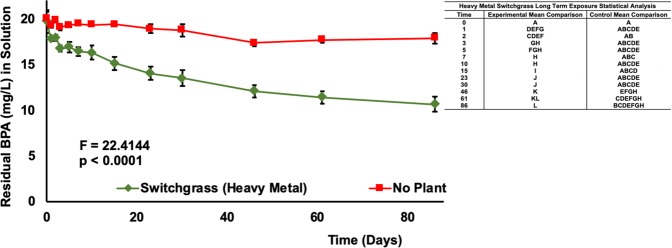
Residual BPA (mg/L) in Aqueous Media at 20 mg/L Initial BPA Concentration over a Period of 86 Days with Switchgrass (Heavy Metal). Data are expressed as mean (n = 10) ± one standard deviation. Mean comparison was carried out by Tukey-Kramer HSD Test and shown in the indented table.

Two switchgrass varieties were tested for their ability to remove BPA (20 mg/L) from aqueous media in a greenhouse setting. Both generic and heavy metal switchgrass varieties removed a significant amount of BPA in 84 days (p < 0.0001, Fig. 2) and 86 days (p < 0.0001, Fig. 3), respectively, compared to no-plant controls. Bisphenol-A concentrations were reduced by 40.4% in the presence of generic switchgrass and 46.1% in the presence of heavy metal switchgrass. Controls exhibited an insignificant decline of BPA at 5.4% (Fig. 2) and 10.6% (Fig. 3) for generic and heavy metal varieties, respectively.

### Kinetic modeling

**Figure 4:**
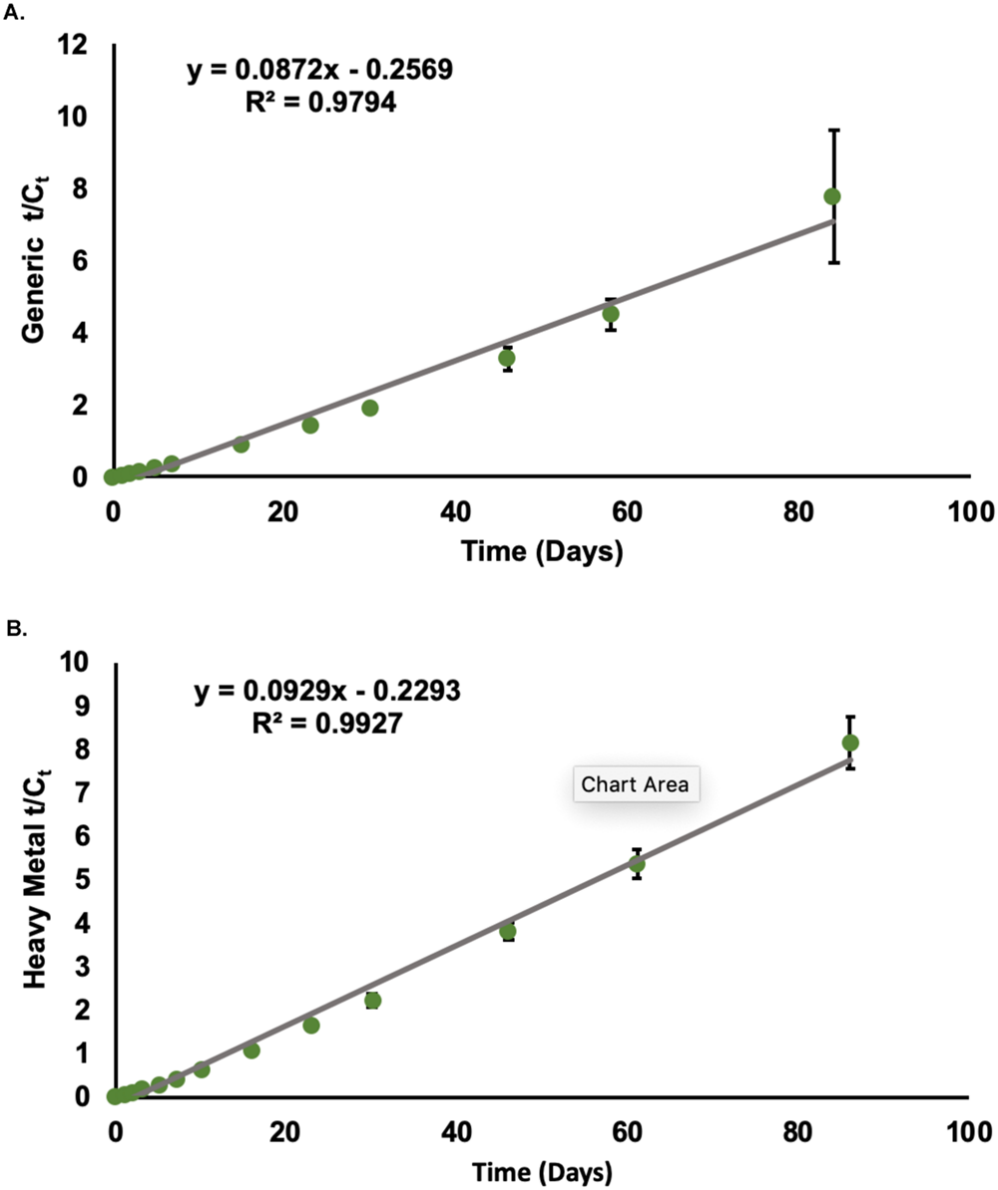
Second Order Fit of BPA Removal by Generic (**A**) and Heavy Metal (**B**) Switchgrass. (**A**) Data are expressed as mean (n = 3). The data points best fit a pseudo-second-order line with an R^2^ = 0.9794. (**B**) Data are expressed as mean (n = 10). The data points best fit a pseudo-second-order line with an R^2^ = 0.9927.

Both varieties of switchgrass demonstrated pseudo-second-order removal kinetics, exhibiting very strong R^2^ values, 0.9794 for the generic variety and 0.9927 for the heavy metal varitey (Fig. 4). Comparison of the initial rate constants (day 0 to day 46) of the two varieties showed the heavy metal variety rate constant was significantly higher (p < 0.001). Although both generic and heavy metal varieties removed a similar fraction of BPA after 84 to 86 days, respectively, the heavy metal variety showed higher initial BPA removal kinetics than the generic one (Supplementary Fig. S1). As evident from the t_25_ and t_40_ values, 25% of BPA was removed from the aqueous solution around day 15 for the heavy metal variety, whereas the generic variety did not reach this level until day 46. Then 40% of BPA removal occurred by day 46 in the heavy metal variety, whereas the generic variety required 84 days to achieve the same level of removal.

### Biochemical analyses

**Figure 5:**
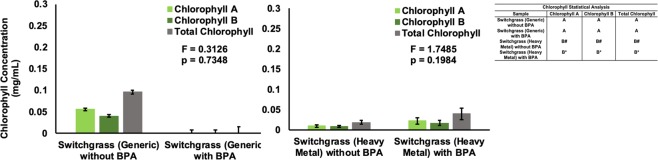
Chlorophyll Content of Generic and Heavy Metal Switchgrass. The graph shows the difference in chlorophyll *a*, *b*, and total chlorophyll in control plants without BPA, and generic and heavy metal switchgrass exposed to 20 mg/L BPA for 84 days and 86 days respectively. Data are expressed as mean (n = 3, *n = 4, ^#^n = 5) ± one standard deviation. Mean comparison was carried out by Tukey-Kramer HSD Test and shown in the indented table. Only the stalk contained a significant amount of chlorophyll.

Phytotoxic effects of BPA on switchgrass were evaluated through chlorophyll content and biomass as a function of BPA exposure. Chlorophyll A, B, and total chlorophyll content were measured after the final day of BPA exposure and compared with unexposed controls. Chlorophyll levels between the controls and BPA-treated plants in both varieties were not statistically significantly different (generic p = 0.7348, heavy-metal p = 0.1984) (Fig. 5).

**Figure 6:**
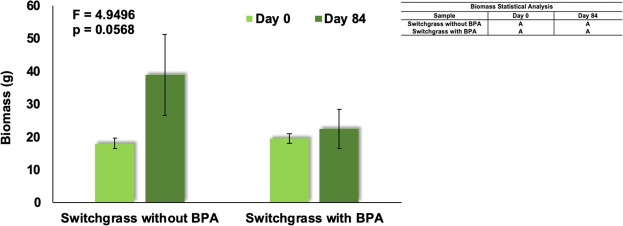
Biomass (g) of Generic Switchgrass as a function of BPA at Day 0 and Day 84. The graph represents the biomass of the switchgrass plants in a control without BPA and in 20 mg/L of BPA before and after the experiment. Data are expressed as mean (n = 3) ± one standard deviation. Mean comparison was carried out by Tukey-Kramer HSD Test and shown in the indented table.

Biomass of generic switchgrass was measured in plants on day 0 and day 84 after BPA exposure and compared with unexposed control plants to evaluate the health of the plant post-BPA exposure. The difference between the controls and exposed plants’ biomass before and after the experimental duration is approaching statistical significance (p = 0.0568). The mean comparison showed an increase in weight for the unexposed controls and the maintenance of a steady weight for BPA-exposed plants over the course of the experiment (Fig. 6). The growth inhibition percentage was 77.9% when generic switchgrass was subjected to 20 mg/L of BPA for 84 days.

**Figure 7:**
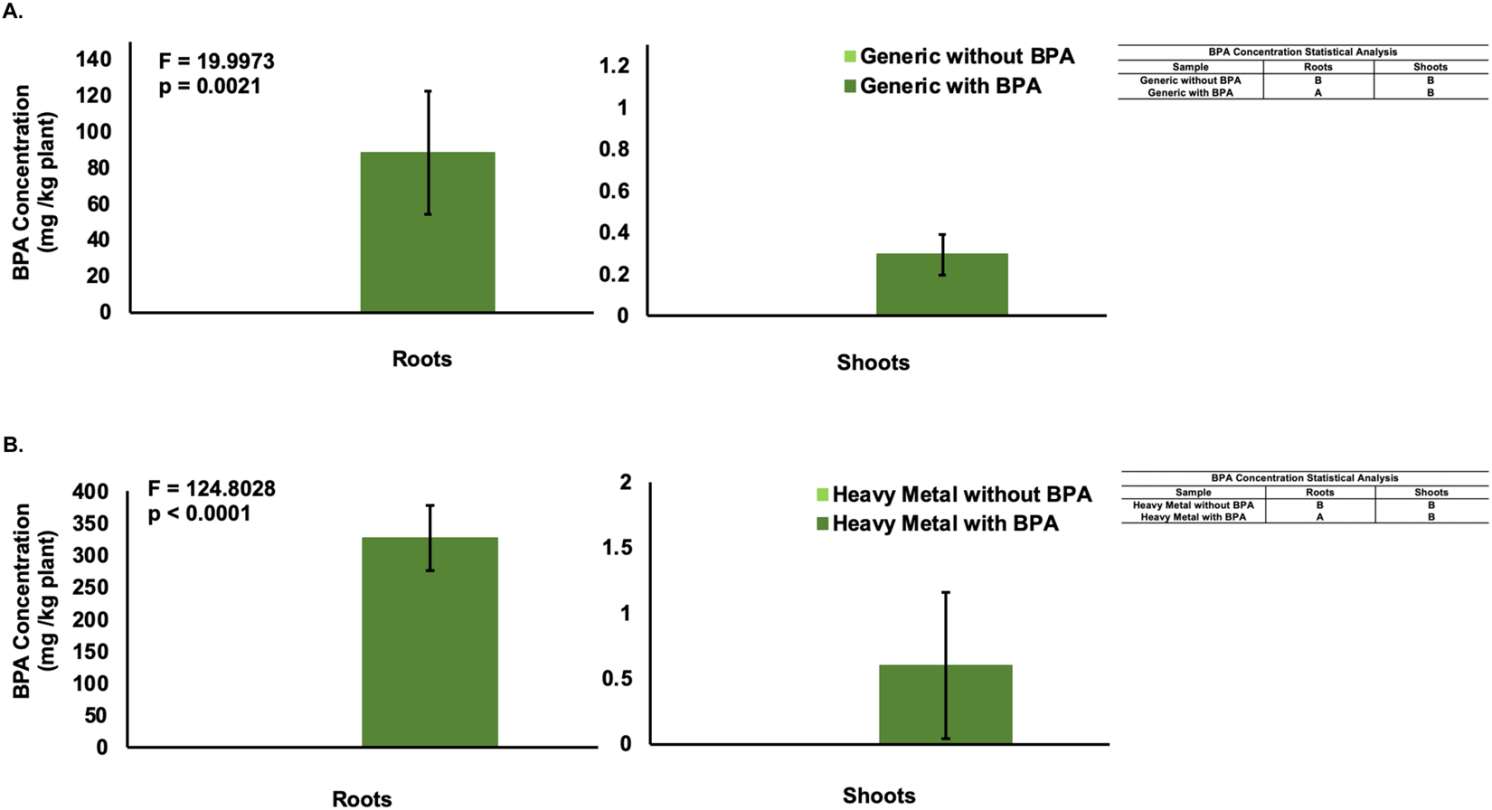
BPA Concentration in Two Plant Tissues (mg/kg) in Generic (**A**) and Heavy Metal (**B**) Switchgrass. (**A**) Data are expressed as mean (n = 3) ± one standard deviation. Mean comparison was carried out by Tukey-Kramer HSD Test and shown in the indented table. (**B**) Data are expressed as mean (n = 3) ± one standard deviation. Mean comparison was carried out by Tukey-Kramer HSD Test and shown in the indented table.

Bisphenol-A accumulation in the plant tissues was significantly higher in the roots as compared to the shoots for both the generic (p = 0.0021) and heavy metal varieties (p < 0.0001) (Fig. 7). Heavy metal switchgrass accumulated significantly more BPA in roots than the generic variety (p < 0.001). No difference was observed in shoot accumulation between either variety (p = 0.3728).

**Figure 8:**
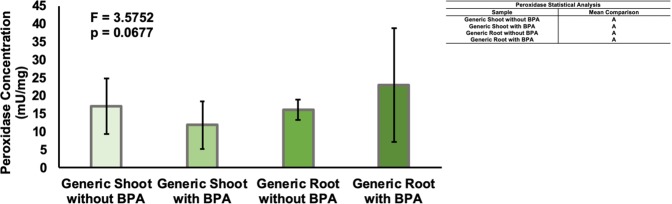
Peroxidase Activity (mU/mg) in Generic Switchgrass as a Function of BPA Treatment. The graph shows the difference in peroxidase level between the plants not exposed to BPA and the plants with 20 mg/L BPA exposure. Data are expressed as mean (n = 6) ± one standard deviation. Mean comparison was carried out by Tukey-Kramer HSD Test and shown in the indented table.

As the mechanism of BPA metabolism might be through the peroxidase pathway, peroxidase activity between BPA-treated and untreated tissues of the generic switchgrass is compared in the roots and shoots. No significant difference (p = 0.0677) was detected between control and experimental plant samples, suggesting the utilization of a different BPA metabolic pathway in switchgrass (Fig. 8). Peroxidase activity in the heavy metal variety was not tested, as the likelihood peroxidase is the method of degradation in one variety of switchgrass and not the other was determined to be minimal.

## Discussion

Bisphenol-A’s persistence and the lack of photolytic and hydrolytic degradation found in the current study supports previous reports, where BPA was found not to be subject to these effects in pure water systems^4,20,21^. Previous studies found BPA degradation under photolytic conditions when humic substances are present, although complete degradation has never been achieved^20,22^. In the presence of humic substances, the half-life of BPA has been reported to be between 66 hours and 160 days, depending on the water conditions, when subjected to photolytic degradation^4^. Without humic substances, the present experiment, concurrent with Im and Loffler’s findings, showed nonsignificant degradation of BPA in photolytic or hydrolytic conditions over 55 days^4^. These findings reaffirm the environmental persistence of BPA and the potential global risk it poses from rising environmental concentrations due to disintegrating plastics in soil, water, and landfills. Studies thus far establish a need to explore sustainable BPA removal technologies from any contaminated media. While phytoremediation shows promise as a sustainable alternative, global application of a BPA-tolerant plant could pose potential harm to the environment if the plant is invasive to the geographical area of implementation. Moreover, using edible plants would increase the risk of exposure owing to bioaccumulation. Thus, native varieties of non-edible plants should be studied on a regional basis to allow for a phytoengineered BPA removal processes to be designed to address the specific needs of the area.

Two varieties of switchgrass, native to the U.S., except for California and the Pacific Northwest, were investigated to evaluate the potential for a phytoengineering technique to remove BPA from disintegrating plastics in soil, water, and landfills to prevent leaching into surrounding environments^40^. Bisphenol-A is still utilized in almost every major plastic production facility in the U.S., though exposure is known to lead to adverse health effects in humans^2^. To date, BPA is found in food containers, medical supplies, and a variety of other products that humans regularly come into contact with^2^. As a result, BPA concentrations in landfill leachate have risen to levels as high as 17 mg/L^18^. With BPA still currently in use, this level will likely continue to increase. Many plants are capable of removing BPA from aqueous media, but none meet the requirements for a phytoengineering technique in the U.S. The most promising plant tested was the broad bean which removed almost all BPA after 21 days when exposed to 50 mg/L^31^. Unlike the broad bean, switchgrass is inedible to humans, decreasing the likelihood of further human BPA exposure. Recent studies have shown a number of photosynthetic organisms native to the U.S. are capable of removing aqueous BPA, including: water velvet (80% removal, 20 days), *Spirogyra* (95% removal, 20 days), and duckweed (96% removal, 20 days), but these aquatic organisms would not survive in soil or landfill environments due to inadequate water supply^32,38^. As a terrestrial, high-biomass, fast-growing, perennial, native grass, switchgrass provides a broader scope of implementation to remove BPA from soil, shallow lagoons, and landfill leachate by creating phytoengineered constructed wetlands surrounding the landfill area.

When analyzing the phytoremediation potential of a plant species, understanding the kinetics of contaminant removal are crucial for designing a phytoengineered remediation process. Both switchgrass varieties showed pseudo-second-order BPA removal from aqueous media. Although the initial kinetics differed between the varieties, the plants showed similar rates of removal after their respective experimental time periods of 84 and 86 days. While a prior study described a kinetic model of the removal of BPA by duckweed and *Spirogyra*^32^, BPA kinetics of removal by a terrestrial plant have not been studied. Establishing kinetic parameters of BPA removal from aquatic media is a positive preliminary step towards the optimization of implementation factors in the phytoengineered BPA removal processes. These parameters will aid in calculating the required plant concentration at given contaminant loads and establishing a balance between plant and BPA uptake. Similar kinetic parameters of BPA removal by switchgrass from soil systems need to be determined and compared with the hydroponic study in future. Moreover, additional investigations of other kinetic parameters of BPA translocation, transpiration, and its biotransformation to other metabolites will provide valuable insight to the phytotolerance of BPA in switchgrass varieties.

Switchgrass showed slower BPA removal rates compared to plants in previous studies providing a probable explanation for the higher BPA tolerance compared to other plants at similar or greater concentrations^31,32,34,36,38,45,46^. Chlorophyll levels, a marker of plant-health, remained unchanged compared to the controls in the BPA-treated heavy metal plants, as opposed to the generic variety, which showed slight phytotoxic symptoms. Other studies assessing the chlorophyll content of algae exposed to BPA, such as *Monoraphidium braunii* and *Stephanodiscus hantzchii*, found these exhibited phytotoxic symptoms^45,46^. Switchgrass withstands the effects of BPA with minimal phytotoxic symptoms, showing promise in a long-term phytoengineering technique.

Previous studies have also compared the change in growth over the experimental duration. Lower concentrations of BPA hinder growth in both *Monoraphidium braunii* (10 mg/L, 4 days) and *Stephanodiscus hantzschii* (15 mg/L, 16 days), with a growth inhibition of 89.23% in the latter^45,46^. Higher concentrations previously tested were found to affect growth in *Chlorella fusca* (Chlorophyta) (18 mg/L and 41 mg/L, 7 days), *Lolium perenne* (perennial ryegrass) and *Raphanus sativus* (radish) (46 mg/L, 16 days), and water velvet (50 mg/L)^38,47,48^. Both perennial ryegrass and radish had approximately 50% less weight than the controls after 16 days^48^. In contrast, the broad bean, one of best BPA-extracting plants reported at present, did not exhibit significant weight difference as compared to the unexposed controls with BPA concentration as high as 50 mg/L after 21 days^31^. Similar results were obtained in the current study, with switchgrass showing minimal phytotoxic effects and nonsignificant biomass degradation at 20 mg/L BPA over 84 days. These results indicate switchgrass has higher BPA tolerance than most other species of organisms tested, potentially due to the detoxification mechanism(s) resulting in higher efficiency of BPA removal from contaminated media.

Analysis of plant tissues showed much higher BPA accumulation in the roots compared to the shoots. The root cells may have a better coping mechanism not present in the shoot cells or BPA translocation to the shoots may have been prevented to avoid further phytotoxicity. Proximity of the roots to the BPA contaminated solution could also contribute to this difference. When contaminants are translocated into the shoot harvesting the plant for proper disposal is easier, but the likelihood of BPA transfer is higher if the plant were eaten. Since switchgrass is sometimes used as a forage crop, animals may eat the shoots, but if BPA accumulation into the shoots is minimal, the risk is low.

Enzymes including peroxidases and polyphenol oxidases in ring cleavage reactions have been implicated as contributors in the break down of BPA^4^. Peroxidase concentration in the roots and shoots of the generic variety switchgrass were measured in an effort to determine a potential mechanism for BPA tolerance. As the peroxidase analysis showed no difference in any tissue type for the controls and BPA-exposed samples, the likelihood this enzyme is responsible for BPA tolerance in switchgrass is low. β-glucosylation has been previously identified as the pathway that metabolizes BPA in Tobacco Bright Yellow-2 cells, which could be an alternative mechanism utlilized by switchgrass^49^. Studies are underway to identify a pathway for BPA metabolism and to identify BPA-induced changes in the metabolomic profile of switchgrass. Alternatively, if a mechanism for degradation cannot be identified within the switchgrass, BPA may be sequestered within the plant tissue. If sequestration is occurring the tissues may be burned to eliminate the BPA. BPA elimination is possible from the sealed burning of plant matter, allowing for the concentrated collection of BPA or complete combustion breakdown of BPA once the plants are removed from the contaminated environment^50^.

Two non-edible varieties of switchgrass, native to most of the U.S., were evaluated as phytoremediation agents capable of extracting BPA from contaminated landfills. Switchgrass, a high-biomass, fast-growing, perennial, native grass, which is environmentally stable and BPA stress-tolerant under long-term exposure, offers a promising phytoremediation strategy^40,41^. Removal efficiencies of both switchgrass varieties were not statistically different, indicating either may be utilized for BPA removal based on availability and cost-effectiveness. The slower initial kinetics of BPA removal in the generic variety may help the plant adapt to BPA-induced stress, resulting in the minimal phytotoxicity symptoms observed at continuous BPA exposure of 20 mg/L over approximately three months. Peroxidase did not show a notable increase in activity in the root and shoot tissues of switchgrass, highlighting the need for further investigation to determine the biochemical mechanisms of BPA tolerance in switchgrass.

Current results are highly promising and have paved the way for an ongoing greenhouse study to fully characterize switchgrass’ potential BPA extraction capacity from soil systems. Once the soil system study is completed, a pilot experiment will determine the feasibility of using switchgrass in the landfill environment. Landfill leachate will be collected and filtered through a constructed wetland where switchgrass is used to reduce the BPA concentration before the leachate is released into the environment. Kinetic parameters will be compared to the current aqueous study, where BPA was fully available for uptake by switchgrass, and the ongoing soil study, where BPA uptake is fractional, relying on the retention and release of BPA as a function of soil properties. The comparative data will provide valuable insights into optimization of a phytoengineering technique to ensure maximum removal of BPA from contaminated landfills and to achieve implementation of this technique in the U.S. to reduce the risk of BPA exposure.

## Materials and Methods

### BPA analysis

An Agilent Technologies (Santa Clara, CA) 1260 Infinity High Performance Liquid Chromatography (HPLC) was utilized to analyze aqueous samples for BPA concentration. The mobile phase of 30% water, 70% acetonitrile was run through an EC-C18 2.7 um Poroshell 120 column (4.6 mm × 50 mm) (Agilent Technologies). Injection volume was 10 μL with a total run time of 4 minutes, analyzed at 228 nm (BPA retention time: 0.806 min). Peaks were manually integrated on OpenLAB CDS ChemStation Edition Revision C.01.05 February 2013 (Agilent Technologies), and a standard curve was generated (0.1 mg/L to 20.0 mg/L), with a quality control (5 mg/L) every nine samples. The limit of detection and quantification were calculated by the standard deviation of the ten lowest standard samples (0.1 mg/L) and the slope from the calibration curve^51^. LOD = 0.005288 mg/L, LOQ = 0.016023 mg/L. Any sample reading lower than the LOQ is reported as < LOQ.

Bisphenol-A concentration in the plant tissue (roots and shoots) after exposure was analyzed by the Environmental Estrogen ELISA kit (Detroit R&D). Samples were rotovapped and diluted with 1X tris buffered saline to the appropriate concentrations. The ELISA was performed as specified in the protocol and analyzed using a Bio-Rad iMark Microplate Reader (Hercules, CA) at 450 nm.

### Hydrolysis and photolysis

Dilutions were prepared from a stock solution of 100 mg/L BPA (Millipore Sigma) in 10% acetonitrile. Solutions of 5 mg/L BPA (0.5% acetonitrile) were produced in three replicates. Hydrolysis was tested by covering one set of flasks with aluminum foil to prevent sunlight exposure. Photolysis was assessed by covering one set of flasks with parafilm. Both sets were placed directly in front of a west-facing window with sunlight exposure. A periodic analysis was conducted over 55 days, with samples filtered through a 0.45 μm PTFE syringe filter (VWR International, Randor, PA) and analyzed on HPLC. Controls were prepared fresh daily.

### BPA removal from aqueous media by *panicum virgatum*

Two different long-term exposure experiments were performed utilizing different varieties of *Panicum virgatum*: generic (Lucas Greenhouses, Fairport, NY) and heavy metal (The Garden Factory, Rochester, NY), one year apart. Heavy metal switchgrass has features of metallic-blue foliage that turns yellow/orange in autumn^52^. Both varieties were grown with soil in a five gallon pot. A greenhouse was utilized to control temperature range (18 °C to 30 °C) and humidity fluctuations (60% to 75%) to simulate an average summer in New York state. The generic variety long-term exposure experiment was conducted in Summer 2016 (n = 3) and the heavy metal variety long-term exposure experiment was conducted in Summer 2017 (n = 10). Plants were acclimatized to half-strength Hoagland’s solution over seven days. Hoagland stock solution (100×) was composed of 13.52 g/L KH_2_PO_4_, 50.6 g/L KNO_3_, 118.0 g/L Ca(NO_3_)_2_·4H_2_O, and 40.0 g/L MgCl_2_·6H_2_O^53,54^. Upon completion of acclimatization, 20.0 g of wet plant biomass were exposed to 1 L of 20 mg/L BPA (in 2% acetonitrile with ½ Hoagland’s Solution as the diluting agent), prepared from the same stock as mentioned above, for 84 days (generic variety) and 86 days (heavy metal variety) respectively. Each plant was placed in a one liter Erlenmeyer flask for the duration of the experiment. Controls included no-plant with BPA solution, no-plant without BPA, and plant without BPA. Over the course of the experiment, samples taken were filtered through a 0.45 μm PTFE syringe filter and analyzed by HPLC, as previously described. An analysis for t_25_ and t_40_ was conducted by identifying the time at which 25% and 40% BPA uptake were achieved. After the study was completed, the weight was recorded for a biomass analysis using a Fisher Scientific Scale SLF303 (Max: 300 g, d = 0.001 g) (Hampton, NH).

### Biochemical analyses

Plants were separated at the base of the shoot, frozen at −80 °C, and pieces of each tissue type were ground using a mortar and pestle while adding liquid nitrogen (Jackson Welding, Rochester, NY) until the tissue was sufficiently frozen. The remaining tissue was stored at −80 °C. Chlorophyll analysis was conducted under minimal light exposure with 0.2 g of plant tissue. A solution of 80% acetone was added to the 0.2 g to obtain a final volume of 2 mL. The solution was vortexed and centrifuged at 10,000 rpm for 15 minutes at 4 °C using an Eppendorf 5424 R (Hamburg, Germany). The supernatant was filtered through a 0.45 μm PTFE syringe filter and absorbance measured at 645 nm and 663 nm on a ThermoFisher Genesys 20 Spectrophotometer (Hampton, NH). Chlorophyll *a*, chlorophyll *b*, and total chlorophyll were calculated according to the equations in Sunkar^55^.

Peroxidase was analyzed with an Invitrogen Molecular Probes Amplex Red Hydrogen Peroxidase Assay Kit (ThermoFisher Scientific, Waltham, MA), according to the manufacturer’s instructions and summarized herein. Ground tissue was combined with assay buffer in a ratio of 0.01 g tissue/500 μL buffer. The tissue solution was vortexed, then centrifuged at 15,000 rpm for 10 minutes using an Eppendorf 5424 R. A volume of 50 μL was removed from the supernatant and analyzed according to the kit instructions. Analysis was performed using a Bio-Rad iMark Microplate Reader at 570 nm.

### Kinetic modeling and statistical analysis

Outlier analysis and two-way ANOVA, for statistical analysis of multiple groups, followed by mean comparisons using Tukey-Kramar for post hocs analysis were conducted with JMP Version 13. Letters within all graphics indicate differences between the means of replicates. Points with the same letter are not statistically different (p > 0.05). All tests were conducted with 95% confidence. Kinetic parameters such as reaction rates for BPA removal from aqueous media were determined following Equation 4 as described by Robati^56^.

## Supplementary information

Supplementary information 

## Data Availability

The datasets generated during and/or analyzed during the current study are available from the corresponding author on reasonable request.
