# Supplementary material for: Phytoremediation potential of switchgrass (Panicum virgatum), two United States native varieties, to remove bisphenol-A (BPA) from aqueous media
Source: Sci Rep. 2020 Jan 21;10:835. doi: 10.1038/s41598-019-56655-w (PMC6972653; doi:10.1038/s41598-019-56655-w)

Title: Phytoremediation potential of switchgrass (*Panicum virgatum)*, two United States native varieties, to remove bisphenol-A (BPA) from aqueous media.

Co-First Authors:

Jacob C. Phouthavong-Murphy^1^, jmurphy1@mail.naz.edu

Alyssa K. Merrill^1^, amerril5@mail.naz.edu

Authors (in order):

Stephanie Zamule^1^, szamule5@naz.edu

David Giacherio^1^, dgiache1@naz.edu

Beverly Brown^1^, bbrown6@naz.edu

Carol Roote^1^, croote7@naz.edu

Padmini Das^1^*, pdas8@naz.edu, phone: 585-389-2552, fax: 585-389-2672

^1^Nazareth College of Rochester. 4245 East Ave, Rochester, NY 14618

**Fig. S.1** Relative Pseudo Second Order Rate of Initial BPA Removal by Generic and Heavy Metal Switchgrass. Data expressed as mean (n=3 for generic, n=10 for heavy metal) + one standard deviation. Mean comparison was carried out by Tukey-Kramer HSD Test and shown in the indented table. The data shown is the initial (until 46 days) pseudo-second-order rate constants of BPA removal (*-k_2_*) by both generic and heavy metal switchgrass.

Figure S.1:


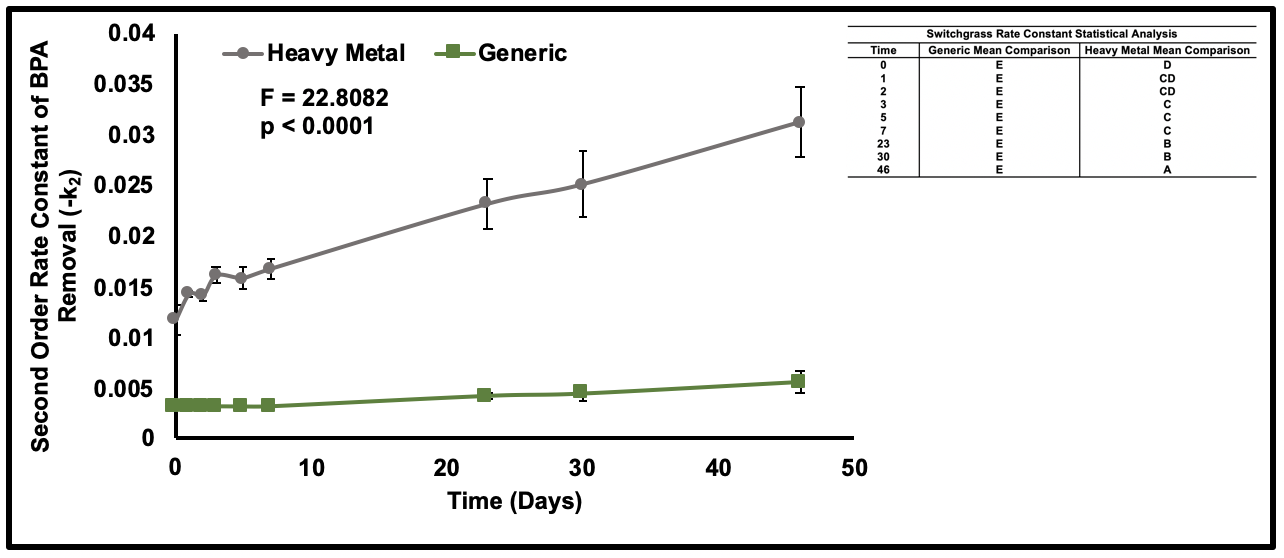

Supplement: Supplementary file 1 — Supplementary information [file 41598_2019_56655_MOESM1_ESM.docx]
